# Notch Signaling in Macrophages in the Context of Cancer Immunity

**DOI:** 10.3389/fimmu.2018.00652

**Published:** 2018-04-09

**Authors:** Tanapat Palaga, Wipawee Wongchana, Patipark Kueanjinda

**Affiliations:** ^1^Department of Microbiology, Faculty of Science, Chulalongkorn University, Bangkok, Thailand; ^2^Center of Excellence in Immunology and Immune-Mediated Diseases, Chulalongkorn University, Bangkok, Thailand; ^3^Institute of Biological Products, Department of Medical Sciences, Ministry of Public Health, Nonthaburi, Thailand; ^4^Laboratory for Systems Pharmacology, Department of Pharmacology, Faculty of Medicine Siriraj Hospital, Mahidol University, Bangkok, Thailand

**Keywords:** Notch signaling, macrophages, tumor-associated macrophages, metastasis, tumor immunity

## Abstract

Macrophages play both tumor-suppressing and tumor-promoting roles depending on the microenvironment. Tumor-associated macrophages (TAMs) are often associated with poor prognosis in most, but not all cancer. Understanding how macrophages become TAMs and how TAMs interact with tumor cells and shape the outcome of cancer is one of the key areas of interest in cancer therapy research. Notch signaling is involved in macrophage activation and its effector functions. Notch signaling has been indicated to play roles in the regulation of macrophage activation in pro-inflammatory and wound-healing processes. Recent evidence points to the involvement of canonical Notch signaling in the differentiation of TAMs in a breast cancer model. On the other hand, hyperactivation of Notch signaling specifically in macrophages in tumors mass has been shown to suppress tumor growth in an animal model of cancer. Investigations into how Notch signaling is regulated in TAMs and translates into pro- or anti-tumor functions are still largely in their infancy. Therefore, in this review, we summarize the current understanding of the conflicting roles of Notch signaling in regulating the effector function of macrophages and the involvement of Notch signaling in TAM differentiation and function. Furthermore, how Notch signaling in TAMs affects the tumor microenvironment is reviewed. Finally, the direct or indirect cross-talk among TAMs, tumor cells and other cells in the tumor microenvironment *via* Notch signaling is discussed along with the possibility of its clinical application. Investigations into Notch signaling in macrophages may lead to a more effective way for immune intervention in the treatment of cancer in the future.

## Introduction

The biological functions of macrophages are diverse and not only limited to their role as the first line of defense during innate immune response. In addition to their protective role against infections, the known roles of macrophages have expanded in recent years, and their involvement in organ development, tissue homeostasis, and metabolic dysfunctions, such as diabetes and obesity, are increasingly appreciated. Cancer is another area in which macrophages have emerged as a crucial player in the creation of a tumor microenvironment that supports tumor growth and metastasis, in opposition to their traditional role as an innate immune cell, whose function is to eliminate cancer cells (1). Therefore, understanding the signaling pathway(s) governing the development, differentiation, activation, deactivation, proliferation, and cell death of macrophages in the context of tumorigenesis is expected to reveal novel strategies for targeting cancer growth more effectively.

The critical functions of the evolutionarily well-conserved Notch signaling pathway in myeloid lineage cell development and, in particular, monocyte/macrophage development are well recognized and have been reviewed extensively elsewhere (2, 3). Recent evidence, using state of the art technologies, revealed better defined subsets of circulating monocytes and the uniqueness and the origin of tissue-resident macrophages (TRMs). This new insight reignited the excitement in the field of macrophage biology. In addition, these studies cast new light and controversy over the origin of macrophages found in tumors, called tumor-associated macrophages (TAMs), and the involvement of TAMs in cancer progression and suppression (4, 5). Within tumors of various origins, macrophages have been observed to accumulate in large numbers and exhibit unique combinations of activated phenotypes (6). In general, TAMs in large quantities are associated with poor disease prognosis, partly by promoting tumor growth, dampening immune responses, and inducing angiogenesis and metastasis (7, 8). Together with the recent advances in the understanding of the roles, Notch signaling plays in the activation and regulation of the immune effector functions of macrophages and in TAMs, these observations have led to the conclusion that Notch signaling is one of the candidate pathways to be manipulated to enhance the host anti-tumor response. In this review, we summarize the current knowledge of the involvement of Notch signaling in macrophage activation, with an emphasis on its role(s) in TAMs. We also discuss the cross-talk among macrophages, tumor cells, and other cells associated with the tumor microenvironment and the potential utility and challenges in manipulating Notch signaling in TAMs for tumor suppression in ways that are beneficial to the host.

### Notch Signaling in Macrophage Activation and Function

The biological functions of macrophages are multi-faceted depending on the external microenvironment, and some functions may be contradictory or opposing to others. For example, during infection or tissue injury, macrophages sense danger *via* various receptors, actively eliminate the source of danger by phagocytosis and chemical mediators, and trigger inflammation by producing inflammatory cytokines to alert other immune cells. After the elimination phase, wounds are healed mainly by anti-inflammatory wound-healing macrophages (9). The contradictory inflammatory and anti-inflammatory microenvironments are conducive to driving macrophage activation into two opposite functional spectra. The most simplistic view of macrophage effector functions divides activated macrophages into pro-inflammatory macrophages, in which macrophages are activated by pathogen-associated molecular patterns (PAMPs) and/or inflammatory cytokines. In contrast, anti-inflammatory macrophages, activated by IL-4/IL-13, represent a wound-healing and immunosuppressive phenotype (10). However, more detailed characterization and studies in various *in vivo* models have revealed a more complicated view of macrophage effector phenotypes that are often observed in an *in vivo* setting (11). Thus, the narrow concept of pro- vs. anti-inflammatory macrophages may be oversimplified, and the presence of various hybrid phenotypes of macrophages has been described (11). Some of the genes uniquely expressed in pro- or anti-inflammatory macrophages are summarized in Table 1 (12, 13).

**Table 1:** Expression profiles of Notch ligands and receptors and some stage-specific makers in tumor-associated macrophages (TAMs).

Notch receptors/ligands or surface markers related to TAMs	Pro-inflammatory macrophages	Anti-inflammatory macrophages	Differentiation stages of TAMs based on study by Franklin et al. (14)				
			Stage 1	Stage 2	Stage 3	Stage 4	Stage 5 (TAM)
CCR2	+	−	+	+	+	+	+
Ly6C	+	−	+	−	−	−	−
CD11c			−	+	+	+	+
MHCII	+	+	−	−	+	+	+
CD11b	+	+^a^	high	high	high	low	low
Vascular cell adhesion molecule1			−	−	−	−	+^b^
CD38	+	−					
Erg2	−	+^c^					

Notch receptors							
Notch1	+	+		+	+	+	+
Notch2	+^d^	+		+	+	+^e^	+
Notch3	+						
Notch4							

Notch ligands							
Jagged1	+						
Jagged2							
Dll1	+	+					+
Dll3							
Dll4	+	+^g^					+^f^

*^a^Italiani and Boraschi (12) provide reviews on murine blood monocyte subsets based on Ly6C expression and their functions in inflammation and tissue repair*.

*^b^Franklin et al. (14) propose TAM markers found in a breast cancer mouse model*.

*^c^Jablonski et al. (13) propose novel markers of M(LPS + IFNγ) and M(IL-4) (CD38 and Erg2) based on gene expression profiles that can exclusively distinguish M(LPS + IFNγ) from M(IL-4)*.

*^d^Palaga et al. (15) report the gene expression profile of LPS-stimulated RAW264.7 macrophages*.

*^e^Ishifune et al. (16) report that Notch receptors are required for CD11c^+^ CX3CR1^+^ macrophage (found in the luminal bed of the small intestine) differentiation, thereby suggesting that Notch1 and Notch2, but not Notch3 may be required for TAM differentiation as TAM is also CD11c^+^*.

*^f^Wang et al. (17) report the Notch gene expression profile in anti-inflammatory-like macrophages isolated from tumors*.

*^g^Bansal et al. (18) report Notch profiles in RAW264.7 M(LPS + IFNγ) and M(IL-4 + IL-13)*.

To avoid oversimplification and confusion over macrophage effector phenotypes, this review will adopt the macrophage nomenclatures proposed by Murray et al. to describe specific macrophage subsets based on the stimuli and effector functions described in each referred study (19). In some instances, where the stimuli were not identified, the microenvironments in which macrophages were described will be used.

Initial reports generally found that Notch signaling primarily operates in macrophages that are activated toward inflammatory functions such as in lipopolysaccharide (LPS)-activated macrophages M(LPS) or LPS in combination with IFNγ M(LPS + IFNγ) (15, 20, 21). Subsequent findings in various pathophysiological conditions also indicated the involvement of Notch signaling in activation and effector functions of pro-inflammatory macrophages (3). Notch signaling, therefore, favors inflammatory macrophages, and when the Notch signaling pathway is pharmacologically or genetically blocked, some of the key pro-inflammatory functions are compromised, including the decrease in the production of pro-inflammatory cytokines, such as IL-6, and the reduction in nitric oxide production (15, 22). To this end, Notch signaling is reported to directly or indirectly influence pro-inflammatory effector functions. Notch signaling can directly regulate transcription of some of the inflammation-induced signature genes, such as *il6, il12b*, and *nos2* (23–25). Using *Rbpj*-deficient mice, Xu et al. demonstrated that canonical Notch signaling tips the effector phenotypes toward inflammatory ones by directly influencing the transcription of a transcription factor IRF8 (22). In addition, Notch signaling also indirectly regulates pro-inflammatory phenotypes through a cross-talk with other signaling pathways, such as NF-κB and mitogen-activated protein kinases (15, 20). Interestingly, metabolic analysis found that Notch signaling supports inflammatory macrophage phenotypes by reprograming mitochondrial metabolism toward oxidative phosphorylation (25). Abrogating Notch signaling in myeloid lineage cells attenuated inflammation in a mouse model of alcoholic steatohepatitis and reduced the severity of endotoxin-induced hepatitis (25). All evidence, therefore, points to a critical role of Notch signaling in macrophage activation toward pro-inflammatory phenotypes in a canonical Notch signaling-dependent (intracellular Notch and CSL/RBP-Jκ-dependent) manner. The question remains whether inhibition of Notch signaling under an inflammatory microenvironment can switch macrophages toward the opposite phenotype, such as anti-inflammatory functions, or whether a lack of Notch signaling only dampens the inflammatory response without directing the macrophages toward other effector phenotypes.

Is Notch signaling dispensable for other types of macrophage effector functions? In macrophages treated with IL-4/IL-13 M(IL-4/IL-13), which normally induces anti-inflammatory macrophages. Notch signaling was long considered to be irrelevant; however, an indicator that Notch signaling is activated in the form of cleaved Notch1 was observed in this condition, albeit with different kinetics than those reported in M(LPS + IFNγ) (26). More importantly, in macrophages with targeted deletion of *Rbpj*, CSL/RBP-Jκ, possibly through canonical Notch signaling, was found to be required for activation of M(IL-4) or M(chitin), including the expression of the gene signature associated with M(IL-4), such as *Arg1* expression (27). This involvement was independent of STAT6, C/EBPβ, and IRF8. In addition, our observation revealed that Notch signaling functions in macrophages activated by PAMPs in the presence of immune complexes and LPS M(LPS + Ic), which predominantly produce high amounts of IL-10 and low levels of IL-12 to function in dampening the immune response (28, 29). Together, these data indicate the need for re-thinking the roles that Notch signaling plays in macrophage activation. Notch signaling may be involved in various types of macrophage activation in a context-dependent manner. Whether Notch signaling functions as an instructor or a signal amplifier during macrophage activation remains to be determined, but this feature is similar to what has been postulated for the involvement of Notch signaling in the polarization of helper T cells (30).

### Notch Receptors and Ligands During Macrophage Activation

Four Notch receptors and five Notch ligands have been identified thus far. Differences in signals sent *via* different combinations of ligand–receptor interactions have long been suspected. For example, two ligands, Dll1 and Dll4, send different signals through the same receptor, Notch1, that are either pulsatile or sustained, thereby inducing different cell fates (31). During macrophage activation, various Notch receptors and ligands have been detected (Table 1). All Notch receptors, except for Notch4, are expressed in pro-inflammatory M(LPS) or M(LPS + IFNγ) (15). Notch3 is selectively upregulated in pro-inflammatory macrophages, such as in M(LPS) and M(LDL) (21). Notch1 and Notch2 are required for differentiation of CD11c^+^ CX3CR1^+^ macrophage subset in the small intestine (16). Similarly, Jagged1, Dll1, and Dll4 are detected in pro-inflammatory macrophages (18). In M(LPS), Foldi et al. reported that Jagged1 is the ligand responsible for autoamplification of Notch signaling in pro-inflammatory macrophages (32). The importance of the Notch-Dll4 axis in pro-inflammatory macrophages was highlighted in a study using blocking antibodies against Dll4. The results revealed that the anti-Dll4 antibody reduced pro-inflammatory macrophage accumulation in inflammatory lesions and attenuated atherosclerosis and metabolic disease (33). Furthermore, during influenza infection, Dll1 expression on macrophages is crucial for dictating the effective anti-viral responses of CD4 and CD8 T cells (34). Nevertheless, knowledge of the effect of specific combinations of Notch receptors and ligands on macrophage activation is still limited, and requires each receptor and ligand to be specifically blocked to evaluate the relevance of different interaction pairs.

### Origins and Functions of TAMs

**Figure 1:**
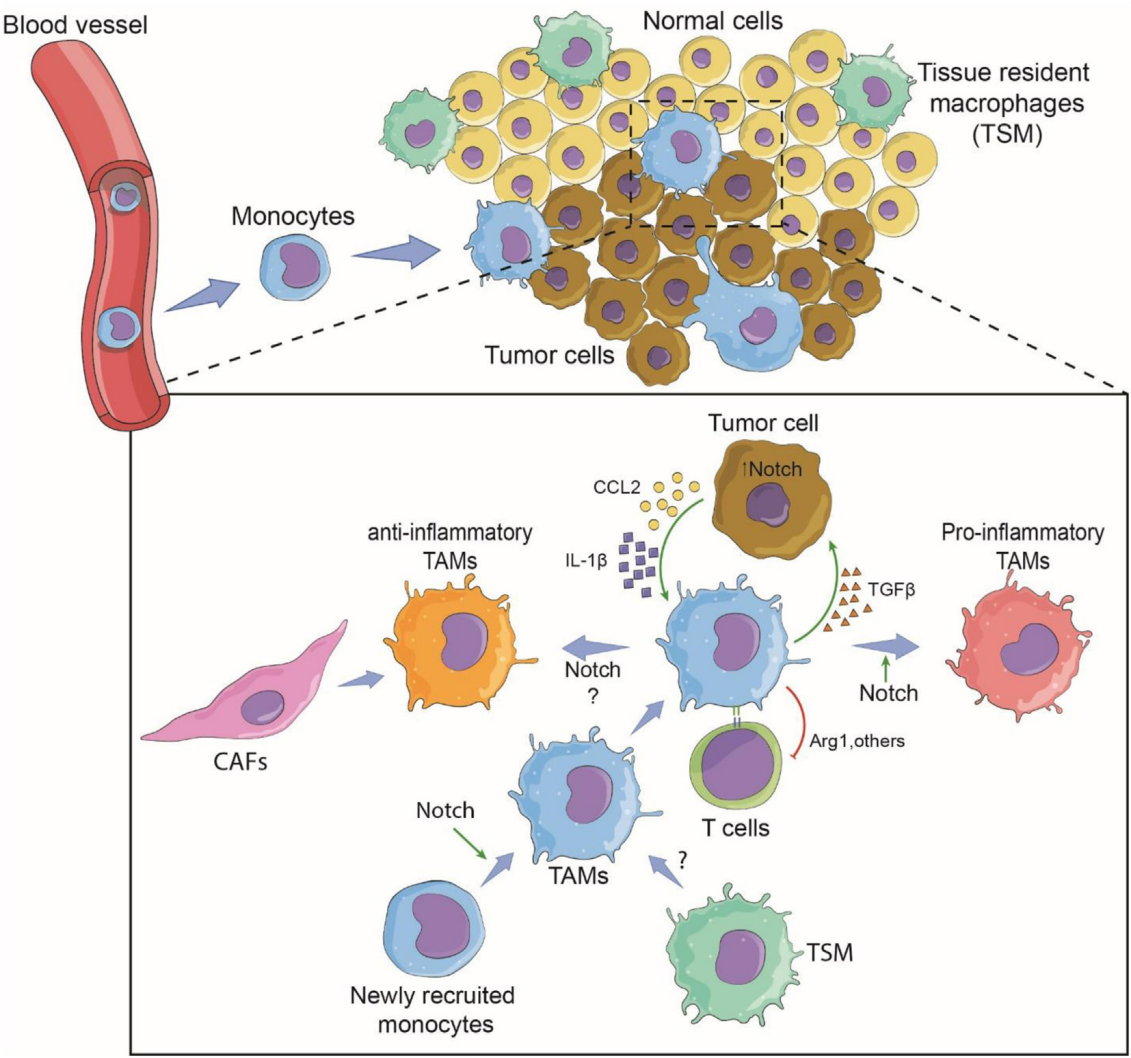
Involvement of Notch signaling during tumor-associated macrophage (TAM) differentiation and tumor growth. Tumor cells recruit monocytes from circulation by secreting chemotactic factors and inflammatory cytokines. Notch signaling may be required for terminally differentiated TAMs. Within the tumor microenvironment, newly recruited monocytes are conditioned to become pro-inflammatory or anti-inflammatory TAMs *via* the cytokine milieu and possibly the canonical Notch signaling (14). Tissue-resident macrophages may also contribute to tumor growth by changing to TAMs. TAMs support tumor growth directly by secreted cytokines and growth factors, and indirectly by affecting T-cell response against the tumor (37). The pro-tumoral function can be Notch signaling dependent or independent. Cancer-associated fibroblasts (CAFs) may also cross-talk with TAMs *via* Notch signaling (38).

In solid tumors, TAMs are a dominant cell type in tumor tissues of various origins, often second to the tumor cells themselves (35). This observation leads to the obvious questions of where these TAMs originate and what are their functions in tumors. There are two potential sources of TAMs. TAMs can develop from newly recruited monocytes from circulation or be derived from TRMs. These sources are not mutually exclusive and depend mainly on the tumor type (5). In a breast cancer model, newly recruited monocytes differentiated to become TAMs, while in brain tumors, both blood-derived monocytes and resident microglia cells contributed to the TAM population (14, 36). When TAMs arise from monocytes recruited from circulation, tumor cells need to secrete factor(s) that trigger the migration of monocytes to the tumor sites (Figure 1).

Macrophage phenotypes, in general, are considered highly plastic and can change depending on the microenvironment, and this may also be true for the phenotypes of TAMs in the tumor microenvironment (10). In one study, human breast cancer cells skewed TAMs toward an anti-inflammatory phenotype partly by secretion of M-CSF (39). In an *in vivo* model of BALB/c 4T1 mammary carcinoma, the tumor microenvironment condition encouraged monocyte precursors to differentiate into diverse TAM subsets with either pro- or anti-inflammatory phenotypes (40). Furthermore, studies in renal cell carcinoma have shown mixed pro- and anti-inflammatory phenotypes of TAMs (41). These observations indicate that there are variations in TAM phenotype that depend on the type of tumors and that the activation of TAMs is highly complex and context-dependent.

### Notch Signaling and TAMs

In TAMs, Notch1 and 2 have been detected in breast cancer model, while Dll1 and Dll4 have been detected in a lung cancer model (Table 1) (14, 17). Jagged1 expression in a breast cancer cell line was shown to modulate TAM differentiation resulting in anti-inflammatory and IL-10-producing TAMs (42). In human cancer, evidence is still lacking regarding the expression profiles of Notch receptors and ligands in TAMs associated with different types of cancer. Recent study of head and neck head and neck squamous cell carcinoma, increasing Notch1 level is associated with CD68^+^/CD163^+^ TAMs, indirectly suggest the link between Notch signaling and TAMs (43). Knowing the expression profiles of Notch receptors and ligands in TAMs and the importance of the signals that they send will provide better targets for intervention.

### Notch Signaling and Migrations of Monocytes and Differentiation Into TAMs

For monocyte-derived TAMs, the presence of TAMs begins with the recruitment of blood monocytes/macrophages to the tumor microenvironment through newly formed blood vessels around the solid tumor (14, 44). Diverse chemokines, i.e., CCL2 (MCP-1), CCL5 (RANTES), CCL7 (MCP-3), CXCL8 (IL-8), and CXCL12 (SDF1), released by tumor cells induce migration, differentiation, and survival of tumor-infiltrating myeloid cells (45, 46). The chemokine receptor CCR2 has been a subject of intense study as a key molecule of monocyte recruitment into tumors. An *in vitro* study revealed that GM-CSF-induced macrophages M(GC) showed higher CCR2 expression than their M-CSF-induced counterparts M(MC). After CCL2 stimulation, M(GC) exhibited enhanced LPS-mediated IL-10 production, indicating an anti-inflammatory role. These phenomena were confirmed by an *in vivo* study in which *Ccr2*-deficient bone marrow-derived macrophages displayed profiles indicative of inflammatory macrophages (47). In the MMTV-PyMT mammary tumor model, a decrease in the number of TAMs in the tumor site was observed in *Ccr2*-null background animals, suggesting the importance of CCR2/CCL2 signaling in the recruitment of TAMs to tumor sites (14). Further investigation revealed that the deletion of *Rbpj* in macrophages results in loss of CCR2 and TAM markers, suggesting a cross-talk between canonical Notch signaling and the CCR2/CCL2 signaling pathway in TAMs in the tumor microenvironment. One can speculate that in the early phase, monocytes are recruited to the tumor site in a CCR2-dependent manner and perhaps begin to encourage activation toward an inflammatory phenotype, but tumor cells educate these cells by creating a tumor microenvironment that re-directs them toward a tumor-friendly phenotype in a later phase of tumor growth (Figure 1). In fact, a gradual increase in M(IL-4)-associated markers such as a high level of CD206 expression and low or no MHC Class II molecule expression has been reported in TAMs in a mouse colon cancer model and in human cancer samples (37). Interestingly, expression of the immune checkpoint receptor, programmed cell death protein 1 (PD1), was significantly increased in CD206^+^ TAMs compared to the expression in TAMs negative for CD206.

In basal-like breast cancer, tumor cells secrete both CCL2 and IL-1β in a Notch-dependent manner, and the secreted cytokine/chemokines, in turn, recruit monocytes to the tumor site (48). In this case, canonical Notch signaling directly regulates the expression of CCL2 and IL-1β, leading to the adhesion of monocytes to blood vessel and extravasation to migrate toward tumor tissue. CCL2 can be produced by bone marrow-derived stromal cells or tumor cells, while tumor cells produce IL-1β (49). Once monocytes are recruited, tumor microenvironments train/educate monocytes to differentiate to become TAMs with a pro-tumor phenotype that can function to support tumor growth and metastasis (5). In this breast cancer model, TAMs interact with cancer cells *via* TGFβ to potentiate the expression of Jagged1, one of the Notch ligands (48). The Notch/Jagged1 positive feedback loop amplifies cytokine/chemokine secretion leading to more TAM recruitment. In an animal model of breast cancer using MMTV-PyMT mice, Franklin et al. showed conclusively that TAMs are recruited from blood inflammatory monocytes and exhibit phenotypes and functions that are distinct from mammary TRMs. Importantly, the terminal differentiation of these TAMs from monocytes is CSL/RBP-jκ-dependent, indicating that the canonical Notch signaling pathway plays a vital role in TAM differentiation (14). Therefore, at least for TAMs in this breast cancer model, Notch signaling plays both an extrinsic role, i.e., regulating the production of recruiting factors by tumor cells, and an intrinsic role, i.e., regulating the differentiation of TAMs. Whether TAMs associated with other tumor types also require CSL/RBP-jκ for their differentiation or function is still an open question.

### Notch Signaling in Anti-Tumor Responses of TAMs

Forced activation of the Notch receptor in TAMs in a Lewis lung carcinoma cell (LCC) model of cancer was shown to repress tumor-promoting activity by enhancing the anti-tumor phenotype and suppressing the pro-tumor phenotype. The mechanism of anti-tumor activity is reported to be mediated in part by microRNAs (miRNAs) (50). miRNAs are small regulatory non-coding RNAs of 21–22 nt that play important roles in regulating gene expression through post-transcriptional silencing of targets mRNAs. miRNAs play important roles in the activation and effector function of macrophages in TAMs by regulating their target genes and signaling pathway (51). In the LCC model, miR-152a, which is under regulation by Notch signaling, targets factor-inhibiting hypoxia 1 and IRF4, a transcription factor involved in M(IL-4) activation, to enhance the anti-tumor phenotype (52). In addition, another miRNA downstream of Notch signaling, miR-148a-3p, also helps to skew the activation of macrophages toward the anti-tumor phenotype by targeting the PTEN/Akt pathway and activation of the NF-κB pathway (53). This observation is consistent with the role of Notch signaling in favoring anti-tumor macrophage activation, and by forced activation of the Notch signaling pathway, these processes can result in the suppression of tumor growth.

Targeted deletion of *Rbpj* in macrophages resulted in reduced activity of CD8^+^ T cells by diminishing the cytotoxic activity against tumor cells in a B16 cell melanoma model (17), suggesting that the cross-talk between TAMs and CTLs is crucial for the anti-tumor immune response, and Notch signaling plays an important role in eliciting the anti-tumor activity of CTL. Moreover, activation of Notch signaling in macrophages was demonstrated to increase the CD8^+^ T cell population infiltrating the tumor site in the LCC model (50). These data indicate the ability of Notch signaling in TAMs to increase anti-tumor activity directly as pro-inflammatory macrophages or indirectly *via* cytotoxic T cells.

With the use of the opposite approach, manipulating canonical Notch signaling in TAMs in a mouse model of cancer was clearly demonstrated to be able to control tumor growth. Targeted deletion of *Rbpj* in macrophages resulted in anti-inflammatory phenotypes under pro-inflammatory inducers (such as LPS), and these macrophages lost the ability to control tumor growth (17). Therefore, if the Notch signaling pathway is dampened in TAMs, this dampening probably results in TAMs shifting toward an anti-inflammatory-like phenotype and helping tumor growth. One caveat is that this study employed *in vitro-*activated macrophages mixed with a tumor cell line that was administered to mice. Whether switching the Notch signaling on or off in TAMs after differentiation in the tumor influences the anti-tumor immunity remains an open question.

Contradictory to the studies described above, several reports have indicated that activation of Notch signaling supports anti-inflammatory phenotypes of macrophages and possibly favors TAMs (27, 54). A study in breast cancer patients who exhibited resistance to aromatase inhibitor treatment showed higher expression of Jagged1 in the tumor and an increasing density of anti-inflammatory TAM infiltration in breast cancer tissue compared to that in control (42). This study indirectly suggests that Jagged1 on cancer cells may drive TAMs into pro-tumor phenotype by activating Notch signaling in TAMs. These contradictory reports on Notch signaling in TAMs imply that the difference in TAM phenotype possibly depends on the tumor microenvironment and types of tumor, and this need to be taken into consideration. In addition, different Notch ligands may activate Notch signaling in different ways, and this may impact the phenotypes of TAMs.

### TAMs, Tumor Angiogenesis, and Notch Signaling

Angiogenesis requires contact between macrophages and endothelial cells together with cytokines and angiogenic molecules. Inflammatory macrophages, including TAMs, are involved in angiogenesis based on the expression of cytokines, such as TNF-α and IL-6, and angiogenic factors, such as vascular endothelial growth factor (VEGF) (5). Because Notch signaling, directly or indirectly, regulates the expression of genes involved in angiogenesis, such as VEGFR and EphrinB2 (55), Notch signaling in TAMs may regulate tumor angiogenesis. In retinal choroidal neovascularization (CNV), the deletion of *Rbpj* in myeloid cells results in the inhibition of the inflammatory response in the retina and choroid after injury. This inhibited inflammatory response is accompanied by suppression of VEGF and TNF-α production and CNV development in the choroid (56). Moreover, Notch1-expressing macrophages interact with two Dll4-expressing sprouts of endothelial cells, leading to the activation of Notch signaling in macrophages. This interaction regulates the function of macrophages during vessel anastomosis in retina angiogenesis (57). Loss of Notch1 in myeloid lineage cells reduces microglia recruitment and results in abnormal angiogenesis (58).

Vascular cell adhesion molecule (VCAM) 1 is highly expressed in TAMs, whereas loss of VCAM1 in macrophages reduces the number of hematopoietic stem cells in the spleen and the inflammation in atherosclerosis due to an inability of macrophages to attach to vascular endothelial cells (59). Although little is known about the role of Notch signaling in the regulation of VCAM1 expression in macrophages, lung endothelial cells express high levels of VCAM1, and increased numbers of TAMs have been observed in lung cancer tissue compared to that in control. Endothelial cells were reported to undergo cellular senescence after implantation of tumor cells expressing Notch ligands (Dll4 and Jagged1), suggesting that VCAM1 expression in endothelial cells is under the regulation by Notch signaling and, together with Notch activation, required for TAM localization (60). VCAM1 expression in endothelial cells is under regulation of the Notch signaling pathway even in the absence of inflammatory cytokines. However, in the presence of IL-1β, VCAM1 expression in endothelial cells is greatly enhanced in a Notch-dependent manner (61). These studies suggest that endothelial VCAM1 is important for the survival of TAMs in the tumor microenvironment. However, this interaction through VCAM1 may be bidirectional because VCAM1 is also highly expressed in TAMs, suggesting that it may play an important role in the survival of endothelial cells as well. Blood vessel endothelial cells have also been found to play a role in TAM differentiation. A recent study demonstrated that Dll1 expressed by endothelial cells lining the blood vessels in mice induced conversion of Ly6C^hi^ to Ly6C^lo^ monocytes in a Notch2-dependent manner (62). This study was the first to demonstrate that the Notch ligand Dll1 in the blood vessel can induce phenotypic changes in monocytes through the Notch2 receptor under steady-state conditions.

### The Role of Notch-Dependent TAMs in Supporting Tumor Growth and Immune Suppression

As described above, TAMs can directly support tumor growth by secreting factors, such as TGFβ (48). TAMs also affect the overall anti-tumor immunity mounted by other immune cells, such as T lymphocytes, in tumor sites by dampening the immune functions. Arginase 1, an arginine-degrading enzyme produced by M(IL-4), can suppress CTL activity (63). Recently, anti-inflammatory macrophage-like (CD206^+^ MHC II^low^ or ^negative^), but not pro-inflammatory macrophage-like (CD206^−^ MHCII^hi^) TAMs have been reported to express PD1 in both a mouse model and in human cancers over time with disease progression (37). The so-called immune checkpoint inhibitor is used to block this PD1-PD-L1 interaction and trigger a vigorous host immune response against the tumor. Interestingly, blocking this interaction results in increasing phagocytosis by macrophages and a reduction in tumor growth in mouse models of cancer (37). Although there is no evidence linking Notch signaling and PD1 in TAMs, there is a report indicating that canonical Notch signaling regulates the expression of PD1 in activated CD8^+^ T cells (64). Cancer-associated fibroblasts (CAFs) are indicated as accomplices in malignant cancers (38). Because CAFs and TAMs are reported to collaborate *via* cell–cell interaction in promoting tumor progression (65), it is possible that Notch signaling may contribute in the cross-talk between the two cell types. Taken together, these observations suggest that Notch signaling may be involved in regulating this immune suppression mechanism in TAMs *via* an immune checkpoint inhibitor.

### Challenges and Potential for Manipulating Notch Signaling in TAMs for Therapy

Notch signaling clearly plays important roles in TAMs, either to promote or suppress tumor growth. Therefore, Notch signaling in TAMs can be a drug target for manipulating host anti-cancer immunity. If Notch signaling in TAMs is pro-tumoral, suppressing it would benefit the host. In contrast, if TAMs require Notch signaling to become more inflammatory anti-tumor macrophages, it needs to be stimulated. Various types of gamma-secretase inhibitor that is a pan-Notch signaling inhibitor are often used to suppress Notch signaling in cancer clinical trials (66). Unfortunately, this inhibitor has off-target effect and is highly toxic if applied systemically. Therefore, designing a method that specifically inhibits Notch signaling in TAMs is desirable. One approach is to use a stapled peptide derived from part of mastermind-like protein that interferes with canonical Notch signaling. If coupled with a TAM-specific delivery system, this peptide could specifically inhibit Notch signaling in TAMs (67, 68). Antibody-based specific antibody blocking has also been investigated for targeting the ligand-binding domain or the negative regulatory region of Notch receptors (69). To activate Notch signaling to favor inflammatory macrophages, an activating antibody that mimics ligand binding may be used. In any case, an intelligent method that targets TAMs is required to minimize the side effects.

### Remaining Unresolved Questions and Future Directions

Notch signaling in macrophages clearly affects their biological functions both directly and indirectly. Notch signaling also affects TAMs and functions in monocyte recruitment, tumor-mediated training, and angiogenesis. Notch signaling in TAMs is, therefore, an attractive signal to manipulate to promote anti-tumor immunity. Macrophages have been reported to be epigenetically modified by stimuli that contribute to “trained immunity” and “tolerance,” at least *in vitro* (70). If the manipulation of macrophage polarization of TAMs through Notch signaling is to be considered as an alternative for cancer treatment, we must ask whether the epigenetic marks on TAMs imprinted by the tumor microenvironment, created by cancer cells, can be reversed or erased so that TAMs could act to benefit the host.

## Author Contributions

TP is responsible for designing the article concept and scope, reviewing 50% of the content, and conceptualizing the figure. WW is responsible for reviewing 20% of the content. PK is responsible for reviewing 30% of the content and designing the table and part of the scope of the article.

## Conflict of Interest Statement

The authors declare that the research was conducted in the absence of any commercial or financial relationships that could be construed as a potential conflict of interest.

## References

[1] Noy R Pollard JW Tumor-associated macrophages: from mechanisms to therapy. Immunity (2014) 41(1):49–61. 10.1016/j.immuni.2014.06.010 25035953 PMC4137410

[2] Ohishi K Katayama N Shiku H Varnum-Finney B Bernstein ID. Notch signalling in hematopoiesis. Semin Cell Dev Biol (2003) 14(2):143–50. 10.1016/S1084-9521(02)00183-0 12651098

[3] Shang Y Smith S Hu X. Role of Notch signaling in regulating innate immunity and inflammation in health and disease. Protein Cell (2016) 7(3):159–74. 10.1007/s13238-016-0250-0 26936847 PMC4791423

[4] Hoeffel G Ginhoux F. Ontogeny of tissue-resident macrophages. Front Immunol (2015) 6:486. 10.3389/fimmu.2015.00486 26441990 PMC4585135

[5] Biswas SK Allavena P Mantovani A. Tumor-associated macrophages: functional diversity, clinical significance, and open questions. Semin Immunopathol (2013) 35(5):585–600. 10.1007/s00281-013-0367-7 23657835

[6] Franklin RA Li MO. Ontogeny of tumor-associated macrophages and its implication in cancer regulation. Trends Cancer (2016) 2(1):20–34. 10.1016/j.trecan.2015.11.004 26949745 PMC4772875

[7] Andon FT Digifico E Maeda A Erreni M Mantovani A Alonso MJ Targeting tumor associated macrophages: the new challenge for nanomedicine. Semin Immunol (2017) 34:103–13. 10.1016/j.smim.2017.09.004 28941641

[8] Mantovani A Marchesi F Malesci A Laghi L Allavena P. Tumour-associated macrophages as treatment targets in oncology. Nat Rev Clin Oncol (2017) 14(7):399–416. 10.1038/nrclinonc.2016.217 28117416 PMC5480600

[9] Zhou D Huang C Lin Z Zhan S Kong L Fang C Macrophage polarization and function with emphasis on the evolving roles of coordinated regulation of cellular signaling pathways. Cell Signal (2014) 26(2):192–7. 10.1016/j.cellsig.2013.11.004 24219909

[10] Biswas SK Mantovani A. Macrophage plasticity and interaction with lymphocyte subsets: cancer as a paradigm. Nat Immunol (2010) 11(10):889–96. 10.1038/ni.1937 20856220

[11] Mosser DM Edwards JP. Exploring the full spectrum of macrophage activation. Nat Rev Immunol (2008) 8(12):958–69. 10.1038/nri2448 19029990 PMC2724991

[12] Italiani P Boraschi D. From monocytes to M1/M2 macrophages: phenotypical vs. functional differentiation. Front Immunol (2014) 5:514. 10.3389/fimmu.2014.00514 25368618 PMC4201108

[13] Jablonski KA Amici SA Webb LM Ruiz-Rosado Jde D Popovich PG Partida-Sanchez S Novel markers to delineate murine M1 and M2 macrophages. PLoS One (2015) 10(12):e0145342. 10.1371/journal.pone.0145342 26699615 PMC4689374

[14] Franklin RA Liao W Sarkar A Kim MV Bivona MR Liu K The cellular and molecular origin of tumor-associated macrophages. Science (2014) 344(6186):921–5. 10.1126/science.1252510 24812208 PMC4204732

[15] Palaga T Buranaruk C Rengpipat S Fauq AH Golde TE Kaufmann SH Notch signaling is activated by TLR stimulation and regulates macrophage functions. Eur J Immunol (2008) 38(1):174–83. 10.1002/eji.200636999 18085664

[16] Ishifune C Maruyama S Sasaki Y Yagita H Hozumi K Tomita T Differentiation of cd11c+ cx3cr1+ cells in the small intestine requires Notch signaling. Proc Natl Acad Sci U S A (2014) 111(16):5986–91. 10.1073/pnas.1401671111 24711412 PMC4000843

[17] Wang YC He F Feng F Liu XW Dong GY Qin HY Notch signaling determines the M1 versus M2 polarization of macrophages in antitumor immune responses. Cancer Res (2010) 70(12):4840–9. 10.1158/0008-5472.CAN-10-0269 20501839

[18] Bansal R van Baarlen J Storm G Prakash J. The interplay of the Notch signaling in hepatic stellate cells and macrophages determines the fate of liver fibrogenesis. Sci Rep (2015) 5:18272. 10.1038/srep18272 26658360 PMC4677309

[19] Murray PJ Allen JE Biswas SK Fisher EA Gilroy DW Goerdt S Macrophage activation and polarization: nomenclature and experimental guidelines. Immunity (2014) 41(1):14–20. 10.1016/j.immuni.2014.06.008 25035950 PMC4123412

[20] Hu X Chung AY Wu I Foldi J Chen J Ji JD Integrated regulation of toll-like receptor responses by Notch and interferon-gamma pathways. Immunity (2008) 29(5):691–703. 10.1016/j.immuni.2008.08.016 18976936 PMC2585039

[21] Fung E Tang SM Canner JP Morishige K Arboleda-Velasquez JF Cardoso AA Delta-like 4 induces Notch signaling in macrophages: implications for inflammation. Circulation (2007) 115(23):2948–56. 10.1161/CIRCULATIONAHA.106.675462 17533181

[22] Xu H Zhu J Smith S Foldi J Zhao B Chung AY Notch-RBP-J signaling regulates the transcription factor IRF8 to promote inflammatory macrophage polarization. Nat Immunol (2012) 13(7):642–50. 10.1038/ni.2304 22610140 PMC3513378

[23] Wongchana W Palaga T. Direct regulation of interleukin-6 expression by Notch signaling in macrophages. Cell Mol Immunol (2012) 9(2):155–62. 10.1038/cmi.2011.36 21983868 PMC4002803

[24] Monsalve E Perez MA Rubio A Ruiz-Hidalgo MJ Baladron V Garcia-Ramirez JJ Notch-1 up-regulation and signaling following macrophage activation modulates gene expression patterns known to affect antigen-presenting capacity and cytotoxic activity. J Immunol (2006) 176(9):5362–73. 10.4049/jimmunol.176.9.5362 16622004

[25] Xu J Chi F Guo T Punj V Lee WN French SW Notch reprograms mitochondrial metabolism for proinflammatory macrophage activation. J Clin Invest (2015) 125(4):1579–90. 10.1172/JCI76468 25798621 PMC4396469

[26] Boonyatecha N Sangphech N Wongchana W Kueanjinda P Palaga T. Involvement of Notch signaling pathway in regulating IL-12 expression via c-Rel in activated macrophages. Mol Immunol (2012) 51(3–4):255–62. 10.1016/j.molimm.2012.03.017 22463790 PMC3358353

[27] Foldi J Shang Y Zhao B Ivashkiv LB Hu X. RBP-J is required for M2 macrophage polarization in response to chitin and mediates expression of a subset of M2 genes. Protein Cell (2016) 7(3):201–9. 10.1007/s13238-016-0248-7 26874522 PMC4791428

[28] Zhang X Edwards JP Mosser DM. Dynamic and transient remodeling of the macrophage IL-10 promoter during transcription. J Immunol (2006) 177(2):1282–8. 10.4049/jimmunol.177.2.1282 16818788 PMC2643023

[29] Edwards JP Zhang X Frauwirth KA Mosser DM. Biochemical and functional characterization of three activated macrophage populations. J Leukoc Biol (2006) 80(6):1298–307. 10.1189/jlb.0406249 16905575 PMC2642590

[30] Tindemans I Peeters MJW Hendriks RW. Notch signaling in T helper cell subsets: instructor or unbiased amplifier? Front Immunol (2017) 8:419. 10.3389/fimmu.2017.00419 28458667 PMC5394483

[31] Nandagopal N Santat LA LeBon L Sprinzak D Bronner ME Elowitz MB. Dynamic ligand discrimination in the Notch signaling pathway. Cell (2018) 172(4):869–80.e19. 10.1016/j.cell.2018.01.002 29398116 PMC6414217

[32] Foldi J Chung AY Xu H Zhu J Outtz HH Kitajewski J Autoamplification of Notch signaling in macrophages by TLR-induced and RBP-J-dependent induction of Jagged1. J Immunol (2010) 185(9):5023–31. 10.4049/jimmunol.1001544 20870935 PMC3010732

[33] Fukuda D Aikawa E Swirski FK Novobrantseva TI Kotelianski V Gorgun CZ Notch ligand delta-like 4 blockade attenuates atherosclerosis and metabolic disorders. Proc Natl Acad Sci U S A (2012) 109(27):E1868–77. 10.1073/pnas.1116889109 22699504 PMC3390871

[34] Ito T Allen RM Carson WFt Schaller M Cavassani KA Hogaboam CM The critical role of Notch ligand delta-like 1 in the pathogenesis of influenza a virus (H1N1) infection. PLoS Pathog (2011) 7(11):e1002341. 10.1371/journal.ppat.1002341 22072963 PMC3207886

[35] Chanmee T Ontong P Konno K Itano N Tumor-associated macrophages as major players in the tumor microenvironment. Cancers (Basel) (2014) 6(3):1670–90. 10.3390/cancers6031670 25125485 PMC4190561

[36] De Palma M. Origins of brain tumor macrophages. Cancer Cell (2016) 30(6):832–3. 10.1016/j.ccell.2016.11.015 27960082

[37] Gordon SR Maute RL Dulken BW Hutter G George BM McCracken MN PD-1 expression by tumour-associated macrophages inhibits phagocytosis and tumour immunity. Nature (2017) 545(7655):495–9. 10.1038/nature22396 28514441 PMC5931375

[38] Liao Z Tan ZW Zhu P Tan NS. Cancer-associated fibroblasts in tumor microenvironment – accomplices in tumor malignancy. Cell Immunol (2018):S8–8749. 10.1016/j.cellimm.2017.12.003 29397066

[39] Sousa S Brion R Lintunen M Kronqvist P Sandholm J Monkkonen J Human breast cancer cells educate macrophages toward the M2 activation status. Breast Cancer Res (2015) 17:101. 10.1186/s13058-015-0621-0 26243145 PMC4531540

[40] Movahedi K Laoui D Gysemans C Baeten M Stange G Van den Bossche J Different tumor microenvironments contain functionally distinct subsets of macrophages derived from Ly6C(high) monocytes. Cancer Res (2010) 70(14):5728–39. 10.1158/0008-5472.CAN-09-4672 20570887

[41] Kovaleva OV Samoilova DV Shitova MS Gratchev A. Tumor associated macrophages in kidney cancer. Anal Cell Pathol (Amst) (2016) 2016:9307549. 10.1155/2016/9307549 27807511 PMC5078639

[42] Liu H Wang J Zhang M Xuan Q Wang Z Lian X Jagged1 promotes aromatase inhibitor resistance by modulating tumor-associated macrophage differentiation in breast cancer patients. Breast Cancer Res Treat (2017) 166(1):95–107. 10.1007/s10549-017-4394-2 28730338

[43] Mao L Zhao ZL Yu GT Wu L Deng WW Li YC Gamma-secretase inhibitor reduces immunosuppressive cells and enhances tumour immunity in head and neck squamous cell carcinoma. Int J Cancer (2018) 142(5):999–1009. 10.1002/ijc.31115 29047105

[44] Carmi Y Dotan S Rider P Kaplanov I White MR Baron R The role of IL-1beta in the early tumor cell-induced angiogenic response. J Immunol (2013) 190(7):3500–9. 10.4049/jimmunol.1202769 23475218

[45] Mantovani A Allavena P Sozzani S Vecchi A Locati M Sica A. Chemokines in the recruitment and shaping of the leukocyte infiltrate of tumors. Semin Cancer Biol (2004) 14(3):155–60. 10.1016/j.semcancer.2003.10.001 15246050

[46] Sica A Mantovani A. Macrophage plasticity and polarization: in vivo veritas. J Clin Invest (2012) 122(3):787–95. 10.1172/JCI59643 22378047 PMC3287223

[47] Sierra-Filardi E Nieto C Dominguez-Soto A Barroso R Sanchez-Mateos P Puig-Kroger A CCL2 shapes macrophage polarization by GM-CSF and M-CSF: identification of CCL2/CCR2-dependent gene expression profile. J Immunol (2014) 192(8):3858–67. 10.4049/jimmunol.1302821 24639350

[48] Shen Q Cohen B Zheng W Rahbar R Martin B Murakami K Notch shapes the innate immunophenotype in breast cancer. Cancer Discov (2017) 7(11):1320–35. 10.1158/2159-8290.CD-17-0037 28790030

[49] Yumimoto K Akiyoshi S Ueo H Sagara Y Onoyama I Ueo H F-box protein FBXW7 inhibits cancer metastasis in a non-cell-autonomous manner. J Clin Invest (2015) 125(2):621–35. 10.1172/JCI78782 25555218 PMC4319427

[50] Zhao JL Huang F He F Gao CC Liang SQ Ma PF Forced activation of Notch in macrophages represses tumor growth by upregulating miR-125a and disabling tumor-associated macrophages. Cancer Res (2016) 76(6):1403–15. 10.1158/0008-5472.CAN-15-2019 26759236

[51] Self-Fordham JB Naqvi AR Uttamani JR Kulkarni V Nares S MicroRNA: dynamic regulators of macrophage polarization and plasticity. Front Immunol (2017) 8:1062 10.3389/fimmu.2017.01062 28912781 PMC5583156

[52] Satoh T Takeuchi O Vandenbon A Yasuda K Tanaka Y Kumagai Y The Jmjd3-Irf4 axis regulates M2 macrophage polarization and host responses against helminth infection. Nat Immunol (2010) 11(10):936–44. 10.1038/ni.1920 20729857

[53] Huang F Zhao JL Wang L Gao CC Liang SQ An DJ miR-148a-3p mediates Notch signaling to promote the differentiation and M1 activation of macrophages. Front Immunol (2017) 8:1327. 10.3389/fimmu.2017.01327 29085372 PMC5650608

[54] Zheng S Zhang P Chen Y Zheng S Zheng L Weng Z. Inhibition of Notch signaling attenuates schistosomiasis hepatic fibrosis via blocking macrophage M2 polarization. PLoS One (2016) 11(11):e0166808. 10.1371/journal.pone.0166808 27875565 PMC5119780

[55] Kofler NM Shawber CJ Kangsamaksin T Reed HO Galatioto J Kitajewski J. Notch signaling in developmental and tumor angiogenesis. Genes Cancer (2011) 2(12):1106–16. 10.1177/1947601911423030 22866202 PMC3411124

[56] Dou GR Li N Chang TF Zhang P Gao X Yan XC Myeloid-Specific blockade of Notch signaling attenuates choroidal neovascularization through compromised macrophage infiltration and polarization in mice. Sci Rep (2016) 6:28617. 10.1038/srep28617 27339903 PMC4919651

[57] Outtz HH Tattersall IW Kofler NM Steinbach N Kitajewski J. Notch1 controls macrophage recruitment and Notch signaling is activated at sites of endothelial cell anastomosis during retinal angiogenesis in mice. Blood (2011) 118(12):3436–9. 10.1182/blood-2010-12-327015 21795743 PMC3179407

[58] Kangsamaksin T Tattersall IW Kitajewski J. Notch functions in developmental and tumour angiogenesis by diverse mechanisms. Biochem Soc Trans (2014) 42(6):1563–8. 10.1042/BST20140233 25399571

[59] Dutta P Hoyer FF Grigoryeva LS Sager HB Leuschner F Courties G Macrophages retain hematopoietic stem cells in the spleen via VCAM-1. J Exp Med (2015) 212(4):497–512. 10.1084/jem.20141642 25800955 PMC4387283

[60] Wieland E Rodriguez-Vita J Liebler SS Mogler C Moll I Herberich SE Endothelial Notch1 activity facilitates metastasis. Cancer Cell (2017) 31(3):355–67. 10.1016/j.ccell.2017.01.007 28238683

[61] Verginelli F Adesso L Limon I Alisi A Gueguen M Panera N Activation of an endothelial Notch1-Jagged1 circuit induces VCAM1 expression, an effect amplified by interleukin-1beta. Oncotarget (2015) 6(41):43216–29. 10.18632/oncotarget.6456 26646450 PMC4791227

[62] Gamrekelashvili J Giagnorio R Jussofie J Soehnlein O Duchene J Briseno CG Regulation of monocyte cell fate by blood vessels mediated by Notch signalling. Nat Commun (2016) 7:12597. 10.1038/ncomms12597 27576369 PMC5013671

[63] Timosenko E Hadjinicolaou AV Cerundolo V. Modulation of cancer-specific immune responses by amino acid degrading enzymes. Immunotherapy (2017) 9(1):83–97. 10.2217/imt-2016-0118 28000524

[64] Mathieu M Cotta-Grand N Daudelin JF Thebault P Labrecque N. Notch signaling regulates PD-1 expression during CD8(+) T-cell activation. Immunol Cell Biol (2013) 91(1):82–8. 10.1038/icb.2012.53 23070399

[65] Hashimoto O Yoshida M Koma Y Yanai T Hasegawa D Kosaka Y Collaboration of cancer-associated fibroblasts and tumour-associated macrophages for neuroblastoma development. J Pathol (2016) 240(2):211–23. 10.1002/path.4769 27425378 PMC5095779

[66] Ran Y Hossain F Pannuti A Lessard CB Ladd GZ Jung JI gamma-Secretase inhibitors in cancer clinical trials are pharmacologically and functionally distinct. EMBO Mol Med (2017) 9(7):950–66. 10.15252/emmm.201607265 28539479 PMC5494507

[67] Purow B. Notch inhibition as a promising new approach to cancer therapy. Adv Exp Med Biol (2012) 727:305–19. 10.1007/978-1-4614-0899-4_23 22399357 PMC3361718

[68] Moellering RE Cornejo M Davis TN Del Bianco C Aster JC Blacklow SC Direct inhibition of the Notch transcription factor complex. Nature (2009) 462(7270):182–8. 10.1038/nature08543 19907488 PMC2951323

[69] Falk R Falk A Dyson MR Melidoni AN Parthiban K Young JL Generation of anti-Notch antibodies and their application in blocking Notch signalling in neural stem cells. Methods (2012) 58(1):69–78. 10.1016/j.ymeth.2012.07.008 22842086 PMC3502869

[70] Ifrim DC Quintin J Joosten LA Jacobs C Jansen T Jacobs L Trained immunity or tolerance: opposing functional programs induced in human monocytes after engagement of various pattern recognition receptors. Clin Vaccine Immunol (2014) 21(4):534–45. 10.1128/CVI.00688-13 24521784 PMC3993125

